# Abstracts

**Published:** 1979

**Authors:** Harry Wingfield


### Abstracts

### South West Surgical Club Meeting, Bath, 2nd and 3rd November, 1979

Chairman: Mr. Harry Wingfield, F.R.C.S.

### HYPERCALCIURIA AND BRAN

**C. Charlton**

In five years over 300 undoubted, fully investigated stone-formers were examined in Bath, of whom over 100 had hypercalciuria. Lowering the urinary calcium is generally accepted as being a useful measure in patients with hypercalciuria, and sodium cellulose phosphate was used at first. However patients found the powder unpalatable; it is also relatively expensive. It thus seemed unlikely that they would take it for the rest of their lives. Bendrofluazide has also been used for treating hypercalciuria in doses of 2.5 — 10 mgs. daily. It was found that the dose required to return urine calcium excretion to normal varied inconsistently with the initial calcium level. Studies done during World War II on the nutritional value of the ration book diet were concerned in part with the action of whole wheatmeal and bran on binding of calcium in the gut. To bind about 250 mgs. of calcium in the gut per day some 300 mgs. of phytic acid were required, which is contained in about 30 grammes of bran. In effect, this means ingesting 2 dessertspoons full per day which is an effective way of lowering the urinary calcium in about 75% of patients.

Work on the relationship between urinary $\gamma$ glutamyl transpeptidase and crystaluria in stone disease done to see if it was possible to isolate the active stage of formation did not prove to be successful.

### TESTICULAR TUMOUR

**P. Smith**

A 10-year retrospective review of testicular tumours in Bath Health District revealed 31 patients with seminoma, 20 with teratoma and 3 with unusual tumours (lymphoma; metastatic carcinoma; reticulum cell sarcoma). Patients showed typical age distributions, with seminomas occuring in the older age groups, whereas teratomas were mainly confined to patients under 40 years. In 11 a clinical diagnosis of epididymitis was made initially because of pain associated with the swelling. Whilst the majority had a history of less than 6 months, at least 10 patients complained of a swelling of the testicle from between one and 6 years duration. In one the tumour occurred in an undescended testis; in 2 others the tumour occurred in patients who had an orchidopexy after the age of 5 years. Due to unnecessary haste in performing orchidectomy investigations were limited. Only 14 patients had a chest X-ray; 7 had chorionic gonadotrophin studies; 8 had an IVP and only 5 had a skeletal scan. 32 patients, however, had post-operative lymphography, and 2 patients open-staging lymphadenectomy.

Patients were treated by orchidectomy. In 41 patients this was combined with radiotherapy including pelvic and para-aortic lymph nodes up to the level of the diaphragm.

27 of the 31 patients with seminoma were alive at the time of review, 2 dying within a year after having presented with widespread metastases. These findings agree with previous studies. Of the 20 patients with teratoma, 12 died within a year with multiple metastases. The remaining 8 remain well at an average of 7 years after treatment. The tumour markers chorionic gonadotrophin and alphafeto protein should be used as indicators of the presence of metastatic disease and the efficiency of treatment.

In common with other studies, the results of treating seminoma were good, but those with teratoma were depressing. Recent studies suggest a combination of radio- and chemotherapy may improve the results.

### THE CELESTIN TUBE IN THE TREATMENT OF BENIGN OESOPHAGEAL STRICTURES

**J. B. Bristol, M. G. Wilson, N. J. Mortensen, H. T. John**

The high mortality and morbidity associated with resection of advanced benign oesophageal strictures has provoked consideration of alternatives. The Celestin tube has been found useful in such cases either as a temporary indwelling dilator or left permanently in situ. At Southmead Hospital, Bristol and Royal United Hospital, Bath, from 1966 to 1976

Celestin tubes were used in two groups of patients with advanced benign oesophageal strictures:

1. 22 elderly, poor-risk patients (average age 76) in whom intubation alone, via a gastrotomy, provided good symptomatic relief of dysphagia.
2. 11 younger, better-risk patients (average age 63) in whom the tube was used as a temporary indwelling dilator combined with repair of a hiatus hernia.

Following removal at a mean of 5 months post-operatively, 73% of patients remained free of recurrence when followed for 2 years. There were 5 deaths in the first group of patients, but in only one of these might it have been related to tube insertion. There were no deaths in the second group.

### END TO END COLONIC ANASTOMOSIS WITH THE EEA AUTO SUTURE STAPLING GUN IN A GENERAL HOSPITAL

**D. C. Britton, R. A. Bolton, L. Jackson, H. T. John**

Leaks and strictures are serious complications of colonic anastomosis; their incidence can depend upon the experience and ability of the surgeon. The EEA staple gun produces a standard anastomosis with an inverted edge. A trial to compare the incidence of complications was done in three groups of patients undergoing end-to-end left colon anastomosis:

Group One (10 patients) using absorbable sutures and a relieving caecostomy.

Group Two (10 patients) using the staple gun and a relieving caecostomy.

Group Three (10 patients) using the staple gun without faecal diversion.

In Groups Two and Three an improvement in mortality, post-operative hospital stay, and complication rate was shown. Faecal diversion did not affect the outcome. Radiological scanning of the anastomoses demonstrated less deformity in these groups.

We conclude that use of the EEA circumferential stapling device can reduce the complications of colonic surgery. It might allow very low intra-pelvic anastomoses in a proportion of cases.

### GONADOTROPHINS IN THE TREATMENT OF CRYPTORCHIDISM

**W. F. W. Southwood**

Our aim has been to encourage the referal of patients with cryptorchidism for a surgical opinion around the age of 4 to 5 years so that definitive treatment can be carried out before degenerative changes occur which might prevent adequate spermatogenesis at puberty.

In all patients presenting over a year an attempt was made at the initial examination to classify them into those with retractile testes, those with truly undescended testes lying in the path of normal descent, and those with ectopic testes lying in the superficial pouch above and lateral to the external ring. Gonadotrophin therapy (500 units Im twice weekly for 6 weeks) was considered only for those boys whose testes lay along the the pathway of normal descent but could not be palpated below the neck of the scrotum.

12 patients were orginally diagnosed as having unilateral undescended testes; 3 (25%) descended following a course of gonadotrophins. 9 patients had bilateral cryptorchidism; they were particularly difficult to assess due to the obesity and scrotal hypoplasia which were frequently present. In 4 patients, however, both testes descended following a course of gonadotrophin injections. In a further 3 patients one side was descended. In this group, therefore, 11 (61%) out of a possible 18 testes descended and the remaining patients required orchidopexy.

### THE USE OF DOPPLER ULTRASOUND IN THE ASSESSMENT PATIENTS WITH PERIPHERAL VASCULAR DISEASE

**A. R. Turnbull**

Blood flow may be determined from the Doppler shift of ultrasound waves, but the measurement is very complex. The pulsatility index, rise time and transit time can however be determined more easily during simultaneous measurements at the proximal and distal ends of an artery. This has been assessed over the femoro-popliteal segment in 123 patients by comparing the results of Doppler ultrasound with arteriograms. Measurements were also made in 43 asymptomatic patients giving a total of 89 results from control arteries and 228 from patients. Arteriography in the patients showed an additional 63 normal segments, 102 complete blocks and 60 with varying degrees of atheromatous change; 3 had been previously bypassed. The damping factor, which is the ratio of the pulsatility indices at the proximal and distal ends of the arteries, and rise time ratios were plotted against transit time. The results showed that there was a clear distinction between normal arteries and those with a tight stenosis or complete block, but the technique did not discriminate between normal arteries and mild atheromatous changes. The differences were similar when the damping factor or the rise time ratio was plotted against transit time, but the rise time was simpler to calculate.

### BREAST SCREENING

K. Lloyd Williams, Barbara Phillips

Mammography is favoured for screening as it can detect impalpable tumours. Though mammography is considered to have a specificity and sensitivity of 90%, it has a poor predictive value when used to screen a large, asymptomatic population. A means is required therefore for selecting the patients in which most, and ideally all of the cancers will be found. Convential thermography has an even poorer predictive value but, if a computer is used to apply a consistent over-reading, specificity can be increased. If the positive group only is X-rayed, the predictive value of mammography will, in theory, be improved.

To determine whether such pre-selection works in practice, aided by the DHSS, we examined 5,672 women using clinical examination and thermography. 20% were thermally positive and were X-rayed together with women in whom there was a clinical abnormality or strong family history. In all 25% of all the patients were X-rayed. 41 cancers were found giving a pick-up rate of 7.2 per 1,000. This result was similar to other surveys in which the whole sample was X-rayed, and agrees with calculated prevalence rates.

### REFERENCES

1. A History of the Bristol Royal Infirmary. 1917. G. Munro Smith, J. W. Arrowsmith.
2. Medical Education in Britain. R. Milnes Walker. The Rock Carling Fellowship 1965. Nuffield Provincial Hospital Trust.
3. The Report of the Committee of Inquiry into the Regulations of the Medical Profession, A. W. Merrison, 1975, HMSO. Cmnd. 6018.
4. Goodenough Report, Report of the Inter-Departmental Committee on Medical Schools. HMSO. 1944.
5. Memorandum GPRC.1. 1948.
6. Todd Report, Royal Commission on Medical Education, 1965–68, para.195. HMSO. Cmnd. 3659.
7. HM(73)2.
8. BMJ 1962, *1,* 466. Conference on Postgraduate Medical Education.
9. PICKERING, G. 1962. Postgraduate Medical Education: The Present Opportunity and the Immediate Need. BMJ *1,* 421–425.
10. LISTER, J. 1978. The Postgraduate Centre Movement. Update, Oct. p.43.
11. Directory of Postgraduate Medical Centres. CPME, London.
12. HM(67)33.
13. BMJ 1972, *2,* p.547. Hands off Postgraduate Centres, Leading article.
14. Report on Postgraduate Medical Centres. CPME, London.
15. HM(72)63.
16. PM(79)3.
17. BMJ 1964, *1,* p.1262. Nuffield Rotating Internship, Bristol.
18. Royal Commission on the National Health Service, Sir Alec Merrison, 1979, HMSO. Cmnd. 7615.

### A SELECTION OF RECENT ADDITIONS TO THE MEDICAL LIBRARY

ALBINI, B., et al.: Immunopathology of the kidney. (Arnold)
ANGIELSKI, S., et al.: Biochemical aspects of renal function. (Huber)
BARNES, S. E., et al.: Oedema in the newborn. (Pergamon)
BENNON, E., Antique medical instruments. (Sotheby Parke Bernet)
BRASHEAR, R. E. & RHODES, M. L., Chronic obstructive lung disease: clinical treatment and management. (Mosby)
BREWER, G. J., The red cell: proc. of 4th internat. conf. (Progress in clinical and biological res., 21) (Liss)
BUKHARI, A. I., et al.: DNA insertion elements, plasmids and episomes. (Cold Spring Habor Laboratory)
BUNN, H. F., et al.: Hemoglobinopathies. (Saunders)
BURGES, S. H. & HILTON, J., The new police surgeon. (Hutchinson Benham)
CLUYSENAER, O. J. J. & VAN TONGEREN, J. H. M., Malabsorption in coeliac sprue. (Nijhoff).
DEMBROSKI, T. M., et al.: Coronary-prone behaviour. (Springer)
DICKINSON, C. J. & MARKS, J., Developments in cardiovascular medicine. (MTP)
DIETSCHY, J. M., et al.: Disturbances in lipid and lipoprotein metabolism. (American Physiological Society)
DUTHIE, H. L. & WORMSLEY, K., Scientific basis of gastroenterology. (Churchill Livingstone)
ECCLES, J. C., Sherrington: his life and thought. (Springer)
ENKER, W. E., Carcinoma of the colon and rectum. (Year Book Med. Publ.)
FABER, D. S. & KORN, H., Neurobiology of the Mauthner cell. (Raven Pr.)
FIGLEY, C. R., Stress disorders among Vietnam veterans: theory, research and treatment. (Brunner-Mazel)
FILSTON, H. C. & IZANT, R. J., The surgical neonate: evaluation and care. (ACC)
GILLHAM, N. W., Organelle heredity. (Raven Pr.)
GIVENS, J. R., Endocrine causes of menstrual disorders. (Year Book Med. Publ.)
GOMPEL, C., Atlas of diagnostic cytology. (Wiley)
GOODWIN, J. W., et al.: Perinatal medicine: the basic science underlying clinical practice. (Williams & Wilkins)
GOW, J. G. & HOPKINS, H. H., Handbook of urological endoscopy. (Churchill Livingstone)
GUNN, J., et al.: Psychiatric aspects of imprisonment. (Acad. Pr.)
HERZ, A., Developments in opiate research. (Dekker)
HOOD, J. D., Vestibular mechanisms in health and disease. (Acad. Pr.)

